# Author Correction: scGNN is a novel graph neural network framework for single-cell RNA-Seq analyses

**DOI:** 10.1038/s41467-022-30331-6

**Published:** 2022-05-04

**Authors:** Juexin Wang, Anjun Ma, Yuzhou Chang, Jianting Gong, Yuexu Jiang, Ren Qi, Cankun Wang, Hongjun Fu, Qin Ma, Dong Xu

**Affiliations:** 1 Department of Electrical Engineering and Computer Science, and Christopher S. Bond Life Sciences Center, University of Missouri, Columbia, MO USA; 2 Department of Biomedical Informatics, College of Medicine, The Ohio State University, Columbus, OH USA; 3 Department of Neuroscience, The Ohio State University, Columbus, OH USA

**Keywords:** Computational models, Machine learning, Software

Correction to: *Nature Communications* 10.1038/s41467-021-22197-x, published online 25 March 2021.

In Figure 2, panels (a) and (b) were inadvertently swapped. The correct version of this figure appears below. This has been corrected in the HTML and PDF version of this article.



