# Supplementary material for: scGNN is a novel graph neural network framework for single-cell RNA-Seq analyses
Source: Nat Commun. 2021 Mar 25;12:1882. doi: 10.1038/s41467-021-22197-x (PMC7994447; doi:10.1038/s41467-021-22197-x)
Supplement: Supplementary file 2 — Description of Additional Supplementary Files [file 41467_2021_22197_MOESM2_ESM.pdf]

Description of Additional Supplementary Files

File Name: Supplementary Data 1
Description: Synthetic dropout test result of scGNN and other imputation tools

File Name: Supplementary Data 2
Description: Cell clustering result comparison between scGNN and other imputation tools

File Name: Supplementary Data 3
Description: Ablation tests with or without graph embedding

File Name: Supplementary Data 4
Description: Comparison between naïve PCA and PCA with graph embedding

File Name: Supplementary Data 5
Description: Comparison between scGNN and PCA with graph embedding

File Name: Supplementary Data 6
Description: Ablation tests with regulatory signal integration

File Name: Supplementary Data 7
Description: Ablation tests with cluster autoencoders

File Name: Supplementary Data 8
Description: Ablation tests for imputation

File Name: Supplementary Data 9
Description: Choosing K and intensities in clustering on the Klein Dataset

File Name: Supplementary Data 10
Description: Parameter searching in imputation on the Klein Dataset

File Name: Supplementary Data 11
Description: Parameter searching in imputation on the Zeisel Dataset

File Name: Supplementary Data 12
Description: Parameter searching in L1/L2 terms in the imputation

File Name: Supplementary Data 13
Description: CTSRs predicted from IRIS3 using the scGNN imputed matrix

File Name: Supplementary Data 14
Description: SP3 regulated genes in OPC, Astrocyte, and Neuron clusters

File Name: Supplementary Data 15
Description: scGNN CPU time and computing resources

File Name: Supplementary Data 16
Description: Tests on dropout affected benchmarks follow a ZINB distribution
